# Assessment of Binder Modification in Dry-Added Waste Plastic Modified Asphalt

**DOI:** 10.3390/polym16141987

**Published:** 2024-07-11

**Authors:** Soheil Heydari, Nioushasadat Haji Seyed Javadi, Hamid Bayat, Ailar Hajimohammadi

**Affiliations:** School of Civil and Environmental Engineering, University of New South Wales, Sydney, NSW 2052, Australia

**Keywords:** waste plastics, dry process, bitumen modification, polymer modified bitumen, modified asphalt mixture, polyethylene

## Abstract

Plastic production has risen steadily, but recycling rates lag. Researchers are increasingly investigating the use of plastics in road construction, especially in terms of modifying asphalt with waste plastics. The dry process, which involves incorporating plastics into hot aggregates, is increasingly gaining traction as an alternative to the wet process, where plastics are added to hot bitumen. Past studies indicate enhanced asphalt mixture properties with the dry process, but there is debate about the role of waste plastics—whether they should be used as aggregates, fillers, or binder modifiers. This study explores the extent to which dry-added waste plastic modified the binder of the asphalt mixtures. Fluorescent microscopy and scanning electron microscopy revealed the impact of plastic on the binder, while image analysis quantified polymer swelling and dispersion in the binder matrix. It was concluded that when plastics are added to hot aggregates, they will act as binder modifiers. Lower plastic content and reduced polymer crystallinity led to increased polymer swelling and better dispersion in the mixture. This study recommends plastic inclusion of less than 2.5% (by volume) in the dry-added method since high plastic content leads to polymer agglomeration, especially for highly crystalline polymers. Additionally, mixes modified with amorphous plastics exhibited superior workability and performance compared to those modified with crystalline plastics. This study also suggests that using plastics to replace both bitumen and filler can improve cost efficiency, reduce the carbon footprint, and enhance the overall performance of the asphalt mixture.

## 1. Introduction

Plastic has become abundant in nearly all industries, spanning from building and construction to transportation, industrial machinery, consumer products, textiles, and product packaging [1]. Due to the increased demand for this commodity, plastic production has seen an annual growth rate of approximately ten percent since 1950 [2], reaching a staggering 367 million tonnes in 2020 [3]. However, plastic recycling rates are alarmingly low; worldwide, only nine percent of plastic waste is recycled [4].

To boost the recycling of plastic waste, researchers have investigated various applications, including the use of plastics in road construction [5]. Specifically, modifying asphalt mixture with waste plastics shows promising outcomes [6]. Researchers began incorporating waste plastics into asphalt mixtures in the 1990s [7], and subsequent studies have examined all types of waste plastics, with high-density polyethylene (HDPE), low-density polyethylene (LDPE), linear low-density polyethylene (LLDPE), and polypropylene (PP) being the most commonly used plastics [8].

Adding plastics to the asphalt mixture can be accomplished using either dry or wet processes [9]. In the wet process, hot bitumen is mixed with plastic, and the resulting binder is subsequently blended with a hot aggregate at around 180 °C [8,10]. This process has been found to enhance the rutting resistance, temperature susceptibility, and rheological properties of virgin bitumen [5]. However, it requires specialized blending equipment, is time-consuming, and energy-intensive [11]. Additionally, the modified bitumen produced using the wet process must be stored in agitated high-temperature tanks to prevent phase separation [12,13]. Given these challenges, the dry process is being explored as an alternative.

The dry process involves mixing plastics with hot aggregate and subsequently adding hot bitumen to the mixture [5]. In comparison to the wet process, the dry process requires a higher amount of plastic but consumes less energy [5] and does not require any specialized equipment [14] or storage tank. Extensive research has been conducted on the dry process [5], with most studies demonstrating improved rutting resistance, fatigue behaviour, moisture susceptibility, and temperature susceptibility of the asphalt mixture [5].

However, conflicting results have been reported in the literature regarding the role of waste plastics in the mixture. A summary of the studies conducted by other researchers and the reported role of waste plastics in asphalt mixture is presented in Table 1. It remains unclear whether waste plastic particles act as fine aggregates, aggregate modifiers and fillers or they melt and modify the bitumen binder [5], particularly for plastics with melting points lower than the asphalt mixing temperature, such as PP, LDPE, HDPE, and LLDPE.

If plastics act as aggregates, a proportion of the aggregates must be substituted by the plastics. This substitution must be volumetric (not by weight) since the density of the aggregates is 2.5 times more than the density of the plastics [32,33]. Also, considering that the binder absorption is higher in crushed aggregates than in plastics, dry substitution alters the optimum binder content of the mix design [5].

If plastics melt and modify the binder, a proportion of the binder must be substituted by plastics, which can significantly increase the viscosity and stiffness of the binder [5].

In both scenarios, the role of the plastics is of high importance and the asphalt mix design must be re-evaluated accordingly after plastic inclusion. However, current reports in the literature are not conclusive and clear about the role of plastics.

The industry partner of this research (State Asphalt NSW [34]) collaborates with Closed Loop [35] to leverage waste and recycled plastics, predominantly PEs and PP, as asphalt additives to enhance the value of asphalt. Therefore, a clear understanding of plastic’s role in asphalt and the best method for plastic inclusion in the mixture is critical. This research aims to develop knowledge about the role of waste plastics in an asphalt mixture using morphological, rheological, and performance analyses. Polypropylene (PP) and polyethylenes (PEs) have been selected as they are the most commonly used plastic types worldwide [36]. These plastics have a melting point below 180 °C and are highly compatible with bitumen, making them ideal candidates for assessing their effectiveness in terms of asphalt modification. By investigating the role of plastics in asphalt mixtures, this research can lead to more effective and sustainable road construction practices. By understanding the role of plastics in asphalt mixtures, the authors can make informed decisions about the mix design, ultimately contributing to the development of a more resilient and eco-friendly road infrastructure.

## 2. Materials

The bitumen used in the experiments, supplied by Viva Energy Australia Ltd, Sydney, Australia, was grade C170 [37]. The softening point, penetration, and viscosity at 135 °C (Pa·s) of the bitumen were measured to be 44 °C, 64.9 mm, and 0.44 (Pa·s), respectively.

To obtain SMA 10 (stone mastic asphalt with 10 mm maximum aggregate size), coarse aggregate, baghouse, Hanson dust, and hydrated lime (HL) were used in accordance with New South Wales Roads and Maritime Services (RMS) specification for SMA 10 [38]. Coarse aggregates and dust were supplied by Hanson Heidelberg Cement Group, and baghouse filler was collected from State Asphalt NSW’s asphalt plant.

Four different plastic types, high-density polyethylene (HDPE), low-density polyethylene (LDPE), linear low-density polyethylene (LLDPE), and polypropylene (PP), were used in this study. Plastics were supplied by Primaplas Pty Ltd, Sydney, Australia in pellet form. The characteristics of the plastics provided by the suppliers are tabulated in Table 2.

## 3. Research Methodology

### 3.1. Methodology, Mixing Methods and Sample Preparation

The investigation of the effects of waste plastics in asphalt mixtures requires a thorough understanding of the role of each type of plastic in the mixture. Different mixing sequences and mixing conditions can change the role of the plastics [5]. Once the plastics’ role in the mixture (based on different mixing methods) was understood, the most desirable mixing method was selected. Asphalt samples were then prepared using that method and performance tests were conducted on the modified mixtures to understand their effects on the mix performance. Thus, in this study, the authors utilized a three-step investigation as follows:

1st step—Determining whether the plastic particles are incorporated into the binder matrix and finding the best mixing method to do so.

2nd step—Investigating how well different plastics are incorporated into the binder, their swelling rate and dispersion in the matrix and their effects on the binder’s viscosity.

3rd step—Investigating the effects of different plastics on the asphalt performance and mix design.

**Figure 1:**
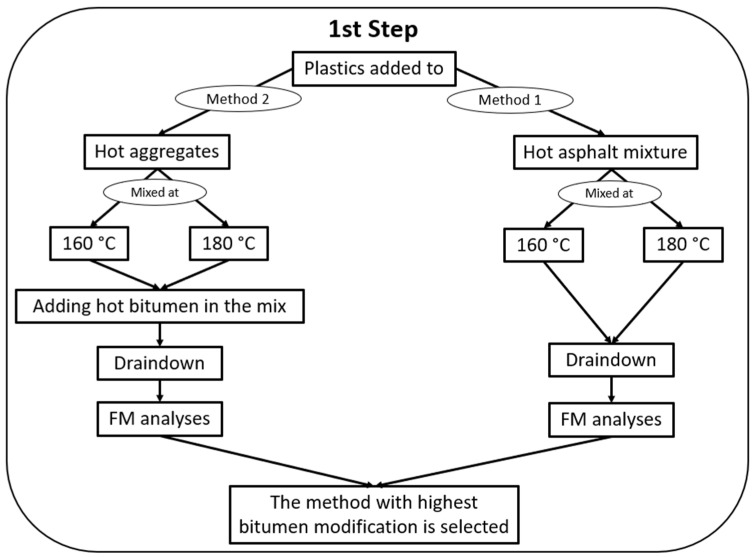
First step of the experiments. The evaluation of the role of plastics in the asphalt mixture using the dry process.

The first step of the investigation is illustrated in Figure 1. In the first step, two methods were used to add plastics to the mixture by dry process. In the first method (Method 1), aggregates and bitumen are mixed, and then plastics are added to the hot asphalt mixture [5,23,25]. In the second method (Method 2), plastics and hot aggregates are mixed, and then hot bitumen is added to the mixture [5,27]. Two mixing temperatures, 160 °C and 180 °C, were used for both methods. The first step of the experiments included a batch of plastics comprising different grades of PE and PP, as tabulated in Table 3.

The authors utilized the draindown test and performed fluorescent microscopy (FM) analyses on the drained binders to detect plastics in the asphalt mixture. The draindown test is described in Section 3.2. Instead of common binder extraction methods, the draindown test was used for binder extraction since those methods, such as centrifuge and reflux extraction, use solvents [39]. The solvents either impact the non-digested plastics in the asphalt mixture and incorporate them into the binder matrix, or the solvent does not digest the plastic, and plastics will not be extracted from the binder. Either way, the common binder extraction method would cause misleading results.

Draindown was performed on the prepared mixes according to RMS T468 [40]. Samples were prepared using the drained binders passing a sieve of 0.15 mm at 160 °C, and a fluorescent microscopy (FM) image was taken of them. This method can show whether the binder is affected by the plastics or not. Table 3 displays the mix design, mixing method, and mixing temperature of each sample.

As will be discussed later in Section 4, the mixing at 180 °C in Method 2 was found to be more efficient in terms of binder modification and was considered to be a more value-adding method compared to Method 1 and other mixing temperatures. Therefore, the specimens for the second step of investigations were prepared via Method 2 at 180 °C.

Polymer-modified binders can be considered as multiphase viscoelastic emulsions [41] in which one phase (or more) is dispersed within the other. Bitumen is a complex mixture of organic compounds that includes asphaltenes and maltene. Asphaltenes are the heaviest and most polar components of bitumen and are often referred to as the “asphaltene fraction” [42]. Maltene is mainly made of resins, saturates, and aromatics and is usually referred to as the non-asphaltene part of bitumen [43]. When plastics are incorporated into bitumen, the polymers tend to swell as they absorb the maltene portion of the bitumen [44]. The resulting swollen polymer is referred to as the polymer-rich phase (PRP), and the rest of the binder matrix is called the asphaltene-rich phase (ARP).

In the second step, the authors compared the plastics with each other to evaluate how well the dry-added plastics modified the binder matrix. Plastic concentrations of 1.25% and 2.5% (total volume of the mix) were considered, as shown in Table 4. Then, the authors analyzed two of the most important parameters: the polymer dispersion in the matrix, and the polymer swelling rate.

**Figure 2:**
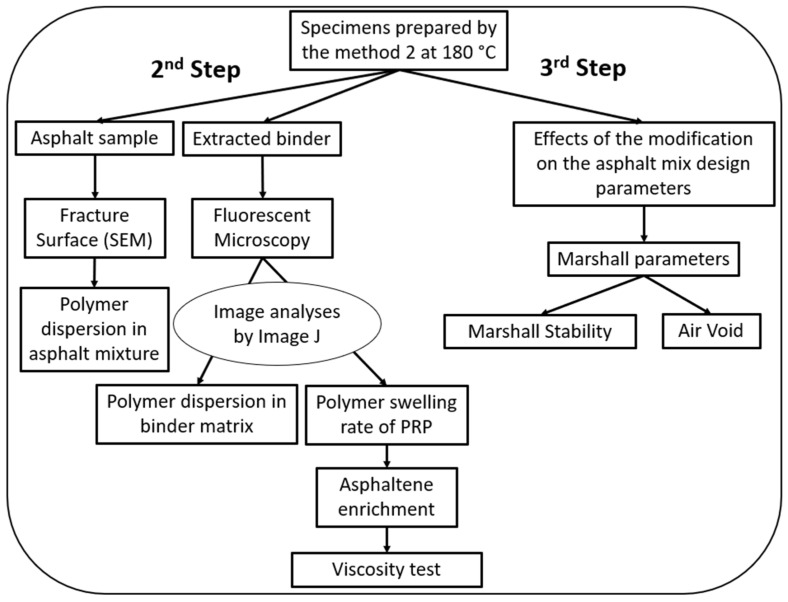
Second and third steps of the experiments for evaluating the effects of the plastics in the asphalt mixture using the dry process.

Higher polymer dispersion implies better homogeneity in the mixture, resulting in a uniform binder property [45,46], and the swelling rate influences how the polymer behaves in the matrix, affecting both the process of making the asphalt mixture and the final quality of the modified asphalt mixture [43]. The swelling rate also defines the volume of the polymer-rich phase (PRP) in the mixture and, subsequently, asphaltene enrichment in the asphaltene-rich phase (ARP). In the second step, the authors also investigated the effects of the swelling rate on the viscosity of the binder, as the most studied viscoelastic property of emulsions [43]. A schematic diagram of the second step is depicted in Figure 2.

Polymer dispersion was examined using two methods: fracture surface analyses and fluorescent microscopy imaging. Fracture surface analyses were performed on asphalt samples to show the dispersion of the polymers in the asphalt mixture alongside aggregates and fillers. This can help to identify polymer interactions with different components in the asphalt mixture. Moreover, fluorescent microscopy was used on the drained-down binder to show the dispersion in the binder matrix. Image J software (bundled with 64-bit Java 8, Windows version) was utilized to analyze the fluorescent microscopy images [47]. Image J was also used to calculate the swelling rate of the PRP, as explained in Section 3.3. The samples and mix designs used in the second step are tabulated in Table 4.

In the third step, Marshall tests were performed on the samples made via Method 2 at 180 °C to understand the effects of the modification on the asphalt mix design parameters, such as air voids and stability. Six different mixtures were prepared, as tabulated in Table 5. Marshall tests were performed on the mixtures. The stability values measured were adjusted in accordance with ASTM 6927, taking into account the volume and thickness of the specimens [48]. At least three Marshall specimens were prepared for each mix design, and the average of the test results was deemed representative of the mixture [48]. The optimum binder content was measured to be 6.8% of the total weight of the mixture (15.65% by the total volume of the mixture). In Runs 2 to 5, 2.5 vol% of the bitumen was substituted by plastics. In Run 6, both the filler (1.25 vol%) and bitumen (1.25 vol%) were substituted by plastic (2.5 vol%).

### 3.2. Marshall and Draindown Tests

The Marshall test is the most commonly employed test to determine asphalt mix design [5]. The Marshall stability and air voids of the samples were measured on the specimens with 2.5% plastic content and on the control sample with no plastics. Air voids are a critical parameter for optimizing asphalt and designing pavements [49]; they also help in predicting the mixture’s performance and workability [5,50]. Marshall stability assesses the ability of the asphalt to tolerate applied loads [51], and Marshall flow is the deformation recorded at the maximum applied load [5]. The Marshall Quotient (MQ) serves as an indicator of the bituminous mixture’s resistance to deformation and is calculated by dividing stability by flow. Calculated MQ values were utilized to assess the specimens’ deformation resistance [25,52,53]. The acceptable ranges for these parameters, as per Australian standards, are tabulated in Table 6.

As outlined in ASTM 6927 [48], Marshall specimens for each mix design were prepared. Specimens were compacted by 50 blows of a hammer at a temperature of around 160 ± 3 °C, as specified in AS 2891 [55]. The draindown test was then conducted for each mix design according to RMS T468 [40]. To conduct the test, the sample was placed in a beaker and covered with a watch glass. Subsequently, the beaker with the sample was placed in an oven at 170 ± 2 °C for 1 h ± 1 min. After the elapsed time, the beaker was removed from the oven, and the sample was promptly transferred into a clean, pre-weighed dish, which could serve as a check measurement. The binder drained from the samples was used for further morphological analysis.

### 3.3. Fluorescent Microscopy

Fluorescent Microscope (FM) imaging was used to investigate the dispersion of the polymers in the binder matrix. The aromatics, with their high concentration of fluorescent benzene rings, are digested into the polymer-rich domains, leading to a higher concentration of aromatics in the PRP compared to the rest of the binder matrix. This phenomenon causes the PRP to appear greener than its surroundings in fluorescent microscopy. [56,57]. Pictures were taken of different polymer concentrations in the mixture to investigate how effectively polymers are dispersed into the binder and to analyze the effects of high polymer content on the polymer swelling rate.

For FM imaging, the samples were prepared using the thin film technique [58]. This involved spreading the samples onto a microscope glass slide, covering them with a cover slip, and rapidly cooling them to room temperature. To ensure accuracy and representativeness for the entire mixture of each sample, ten thin glass slides were produced from each PMB sample [59]. Each sub-sample was then examined using a fluorescent microscopy machine (Olympus vs. 2000, Olympus, Tokyo, Japan) at a magnification of 100× and at room temperature.

Image J software [47] was used for the analysis of the FM images. The concentration of the polymer-rich phases and dispersion variations in different samples were measured. For each mix design, ten samples were taken from the extracted modified binders and the average of the results was considered to be representative of the mixture. The following equation was used to calculate the swelling rate [43]:

(1) Swelling rate=The area of polymer reach phaseThe whole area of the binderthe amount of polymer in the binder matrix%

The term “Variation of PRP Dispersion” was also defined to quantify the variation between PRP dispersions of the same type of polymer in different taken images. The lower variation in PRP dispersion means a more homogeneous binder. This term is calculated using the following equation:

(2) Variation of PRP Dispersion=∑(Area of PRP in the sample−Average area of PRP in all samples)2(Total number of samples−1)

**Figure 3:**
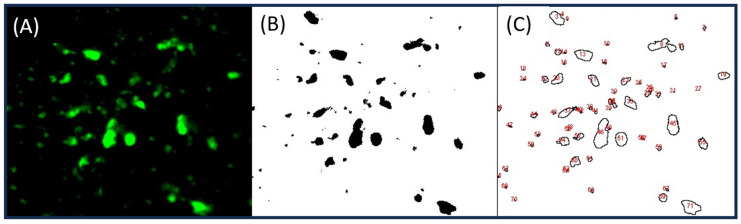
Particle analyses using image j. (**A**) the FM image, (**B**) adjusted threshold in binary format, (**C**) particle analyses, including counts, area, average size, and percentage.

Image J software can be used for particle analysis [47]. First, the image file should be converted to binary format by adjusting the threshold so that only the particles of interest are highlighted, as depicted in Figure 3. The Analyze Particles function can then be used to count and measure the particles [47].

### 3.4. Fracture Surface Analyses Using Scanning Electron Microscope

Fracture surface analysis [60] was used to investigate the microstructure of the asphalt mixes. To do so, a scanning electron microscope (SEM) was utilized in this study [61,62,63,64]. This technique is similar to optical microscopy, but it uses focused electrons to scan the sample and produce high-resolution images [65]. The samples were taken from the asphalt mixtures prepared according to Table 4. The SEM machine utilized in this study was a TM4000Plus (Hitachi, Tokyo, Japan) with an accelerating voltage range of 5–20 kV. To prepare the samples, the specimens were cooled to 5 °C and ruptured from the middle to analyze their fracture surface. A 15 nm gold coating was applied to the samples using the Emitech K550x (Emitech, Casablanca, Morocco, Montigny-le-Bretonneux, France) gold sputter coater. The morphology of the fracture surface was then analyzed by mounting the sample on the sample stands.

### 3.5. Viscosity Test

The viscosity of an asphalt binder is an indication of its resistance to flow [66,67,68]. Plastic inclusion in the bitumen changes the overall viscosity of the binder [5,69], and given that in the dry-process plastics are added straight into the asphalt mixture, their effects on the viscosity of the binder matrix are unknown. To understand their effects on binder viscosity, the authors have modified bitumen with LDPE using the wet process. The selected mixing conditions for the procedure included a mixing temperature of 180 °C, a mixing time of 60 min, and a mixing speed of 1500 rpm, as these have been found to be the most efficient mixing conditions [69]. The rotational viscosity of the binder at temperatures of 165 °C was measured using a Brookfield viscometer in accordance with AS 2341.3 [70].

## 4. Results and Discussion

**Figure 4:**
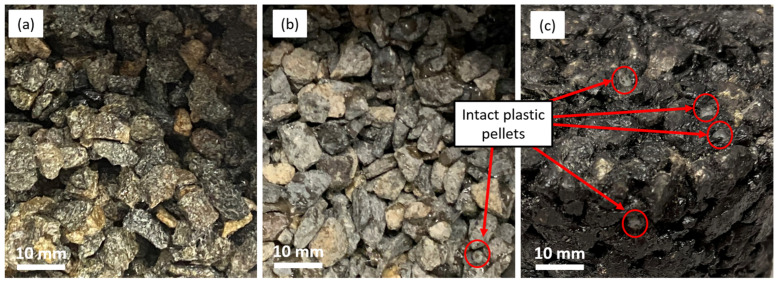
Mixing plastics with hot aggregates. (**a**) mixture b-180 made via Method 2, (**b**) mixture b-160 made via Method 2 and, (**c**) mixture a-180 made via Method 1.

Plastics were added to the mixture using the two most common methods in the dry process (Method 1 and Method 2) [5]. As shown in Figure 4c, the assessment showed that when plastics were added to the hot asphalt mixture (Method 1), plastics were not melted and were almost intact. The plastic particles kept their pellet form and acted as an aggregate in the mix. In this case, a proportion of the aggregate must be substituted by plastics to keep the grading of the aggregate constant. This would not change the grade of the bitumen and would not have any positive effects on the binder properties. Therefore, this method was not considered in this study and excluded from further analyses.

In the second method (Method 2), however, plastic particles were melted and coated on the surface of the aggregates (as shown in Figure 4a,b), indicating that there is no need for the substitution of aggregates with plastics. It was also evident that a mixing temperature of 180 °C was more efficient. As shown in Figure 4a,b, at the higher temperature, plastics perfectly coated the aggregates and formed a shiny layer on their surface. The second step involved studying the behaviour of plastics after adding bitumen to the mixture in the second mixing method.

Draindown samples were taken from mixtures made via Method 2 and were analyzed using their FM images for mixing temperatures of 180 °C and 160 °C, respectively. The green colour observed in the images signifies the plastics swollen by the aromatic components of the bitumen [43].

**Figure 5:**
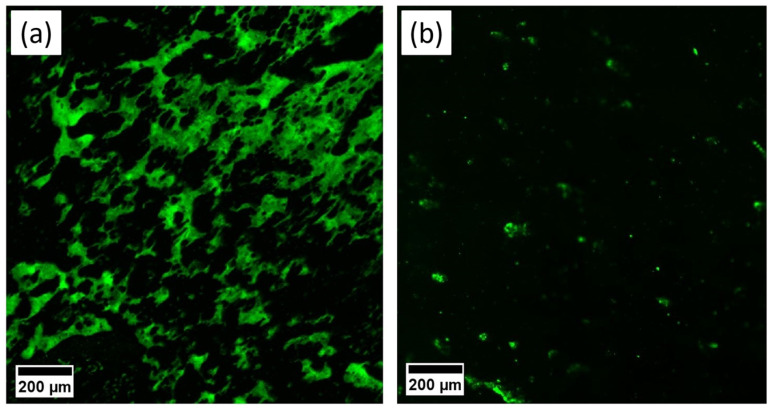
FM images of extracted binders from samples prepared by Method 2. (**a**) mixed at 180 °C (b-180), (**b**) mixed at 160 °C (b-160).

The findings indicated that plastics were effectively mixed with bitumen, with higher mixing temperatures resulting in increased plastic content. As presented in Figure 5b, at a mixing temperature of 160 °C, a small amount of plastic is digested in the binder. However, at 180 °C, plastics were successfully mixed with the binder, resulting in a bitumen modified with a high plastic content. This can lead to the development of a multiphase viscoelastic matrix. Therefore, it is necessary to understand the viscoelastic behaviour of polymers and bitumen in this matrix, as well as the swelling of polymers and their dispersion in the matrix. Understanding these factors is important for predicting and controlling the properties of the resulting material and developing new polymer–bitumen composites with the desired properties.

### 4.1. Swelling Rate and Dispersion of Plastics in the Asphalt Matrix

**Figure 6:**
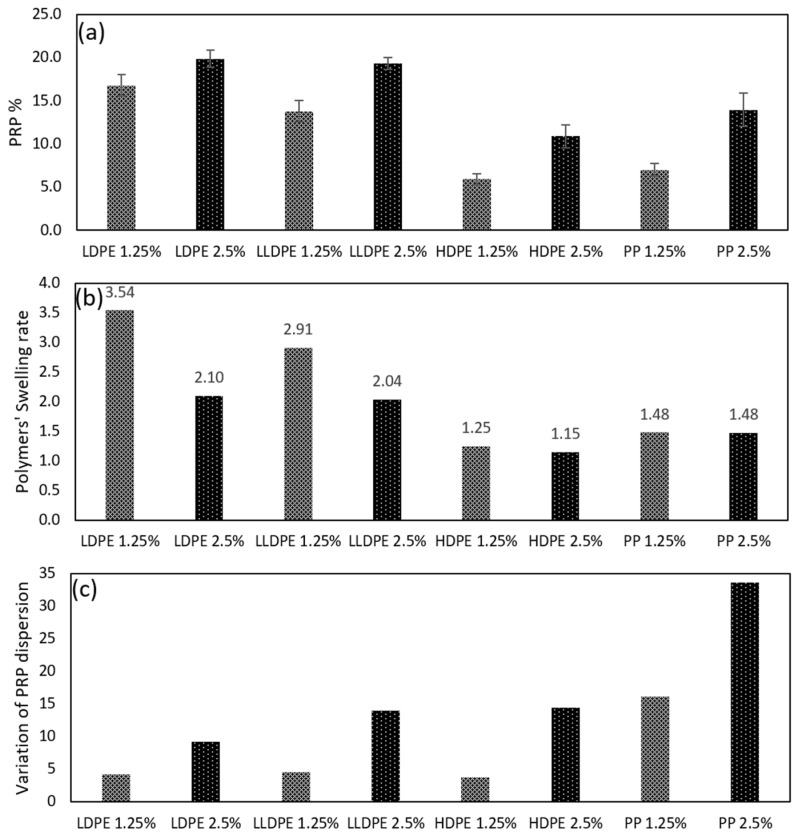
PRP %, polymers’ swelling rate, and variation of PRP dispersion in the matrix. Polymer-rich phase % (**a**), polymers’ swelling rate (**b**), and variation of polymer-rich phase dispersion (**c**).

The authors examined the percentage of polymer-rich phase (PRP) present in the matrix, the swelling rate of the polymers, and the variation of the PRP dispersion in the mixture for each type of plastic. The results of this investigation are presented in Figure 6.

As shown in Figure 6a, in amorphous polymers (LDPE and LLDPE), the percentage of polymer-rich phase (PRP) in the matrix increases slightly when the amount of polymers is doubled. This suggests that the level of polymer swelling at lower polymer contents (1.25% of the total weight of the mixture) is higher than that at higher polymer contents (2.5% of the total weight of the mixture) for these polymers, which is aligned with Figure 6b, where polymer swelling rate is measured using Image J analyses and using Equation (1). 1.25% LDPE and LLDPE swelled to 3.54 and 2.1 times their original volume, respectively, which were way higher than when 2.5% of the polymers were added to the mixture.

In HDPE and PP, as highly crystalline polymers, swelling rates were less affected by the inclusion of more polymers. The reason for this is that since crystalline polymers absorb relatively less aromatics than amorphous polymers [43], there will still be enough aromatics left in the binder for the higher percentages of polymers to absorb and swell.

**Figure 7:**
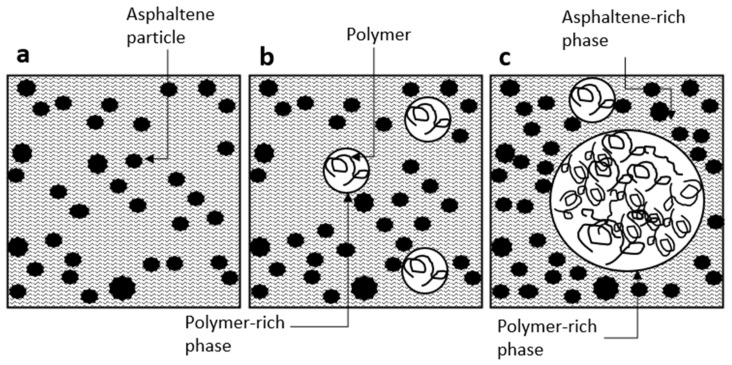
The effect of polymer inclusion on the structure of the binder: virgin bitumen (**a**), low-concentration plastic inclusion (**b**), and high-concentration plastic inclusion (**c**).

As depicted in Figure 6b, the swelling rates for all types of polymers were equal or higher for 1.25% inclusions compared to 2.5% inclusions. The presence of lower polymer content significantly makes a robust swelling effect in the polymer particles due to the abundant number of aromatics accessible to polymer particles within the matrix. Also, in general, low solute (here, polymer) inclusion promotes a strong interconnection with solvent (here, bitumen), while a minor attraction exists between the solute (polymer) particles [71], as shown in Figure 7b. However, as the polymer content increases, both the swelling and interfacial interaction between polymer and bitumen decrease while the attraction between particles strengthens, as shown in Figure 7c. [72].

Among different types of plastics, the highest swelling rate occurred with LDPE, which is around 3.5 and 2 for 1.25% and 2.5%, respectively. It is followed by LLDPE and PP, and the least swelling was observed for HDPE. This difference seems to be due to the crystallinity content of each plastic. In polymers, depending on the swelling agent, swelling occurs within the amorphous or crystalline regions, causing expansion in the polymer [73]. HDPE has the highest crystallinity among the plastics in this study and showed the lowest swelling rate. The trend is followed by PP, LLDPE, and LDPE. This shows that the higher crystallinity of the polymer results in lower swelling rates of that polymer when the solvent is bitumen or, more specifically, when the swelling agents are aromatic components in bitumen.

The other important factor studied here is the variation in PRP dispersion in the binder matrix, which indicates how well the polymer is dispersed throughout the matrix. Higher variations indicate lower homogeneity, meaning that there are areas in the matrix with either very low or very high PRP concentration. Therefore, a lower variation in PRP dispersion is desirable as it leads to a more homogeneous matrix.

**Figure 8:**
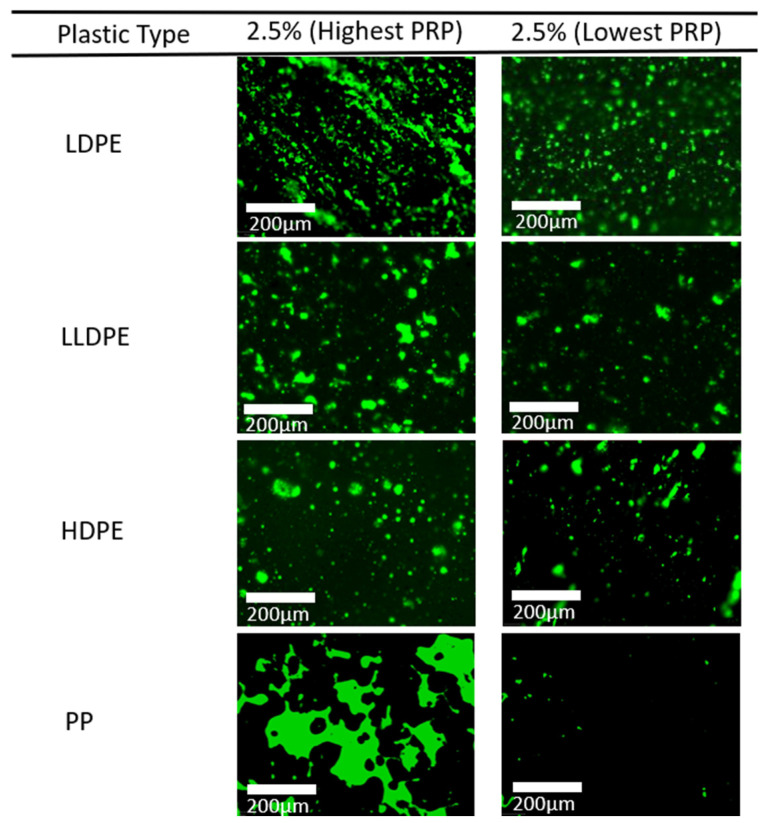
FM images of dry-added plastics in the asphalt mixture. The green area represents PRP, and the black area represents ARP. The highest PRP is the FM image taken from the area in the binder with the highest PRP concentration, and the lowest PRP is the FM image taken from the area with the lowest PRP.

Figure 6c presents the variations in PRP dispersion among different types of plastics, and Figure 8 shows the fluorescent microscopy images of the highest PRP concentrated areas and the lowest PRP concentrated areas amongst all the taken FM images at a 2.5% polymer concentration. Among the polymers, LDPE produces the most homogeneous matrix, followed by LLDPE and HDPE. On the other hand, PP inclusion results in a highly heterogeneous matrix, as shown in Figure 8, which provides a better illustration of the PRP dispersion of different types of plastics in the matrix. The addition of LDPE, LLDPE, and HDPE leads to a homogeneous matrix. However, the inclusion of polypropylene leads to PRP agglomeration in the matrix, particularly at higher inclusion levels (2.5%).

In PP, apart from its crystalline content, melting point plays an important role too. The high melting point of PP, around 160 °C, hinders proper mixing of PP with bitumen and consequently leads the PRPs to agglomerate in one section.

**Figure 9:**
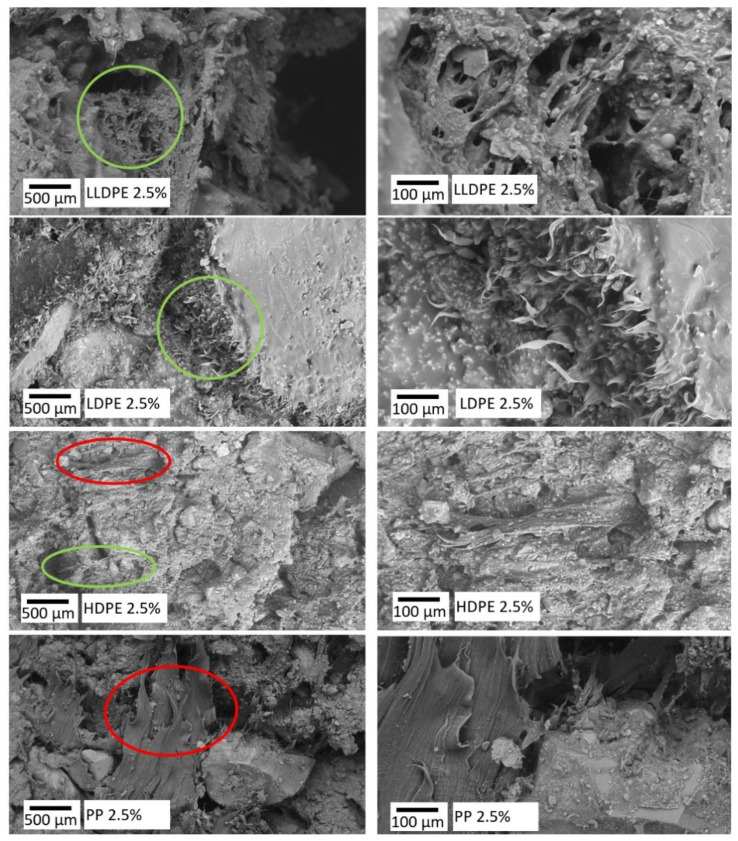
SEM images of the fracture surface of the asphalt mixes containing 2.5% dry-added plastics. Observable examples of good dispersion are circled in green, and observable examples of agglomeration are circled in red. The images on the right display a five-fold magnification of the photographs on the left, offering improved clarity in terms of illustrating the morphology of the circled sections.

As depicted in Figure 6, the highest dispersion is observed with LDPE, with the lowest crystallinity and lowest dispersion observed with PP, which has the highest melting point and high crystallinity. This is also clearly presented in SEM images shown in Figure 9, where LLDPE and LDPE are dispersed in an asphalt mixture, resulting in small elongated-and-dispersed PRPs in the fracture surface (shown as green circles in the images). As shown in Figure 6, the polymer with the highest swelling rate (LDPE) results in less variation in terms of dispersion and, consequently, a more homogeneous matrix due to the similar viscosities between the solvent (bitumen) and the solute (PRP) (which is observable from Figure 9). In other words, the lower crystallinity of the polymer results in a higher swelling rate, lower melting point and viscosity of the formed PRP, and better homogeneity in the bituminous matrix (shown in Figure 9).

The fracture surfaces shown in Figure 9 provide insight into the dispersion of polymers in the asphalt mixture adjacent to the aggregates and filers. All four types of plastics used in this study are clearly washed from the aggregates and cooperated in the binder matrix. This is noticeable since plastics were first mixed with hot aggregates, coated on the surface of the aggregates, and then bitumen mixed with the mixture. The largest polymer agglomeration observed in PP is circled in red in Figure 9. Some polymer agglomerations were also observed in HDPE; however, they are relatively smaller than PPs. LDPE and LLDPE fracture surfaces show the good dispersion of these polymers through the matrix.

### 4.2. Asphaltene Enrichment and Viscosity of the Binder

Particles suspended in viscous material raise the viscosity. Einstein proposed a theory to address this phenomenon. His theory and equation are for when the content of particles is mixed with a liquid, and there are no interactions between the suspended particles [74].

Frankel and Acrivos [17] introduced a model for high-concentration suspensions. They assumed that the average viscosity of a liquid is the viscosity of the suspended particles, neglecting any boundary effects between particles and liquid. However, at very high concentrations, close to maximum concentration, this equation tends to infinity, which cannot be an accurate prediction. Hesami et al. [75] proposed another theory to address very high concentrations. At maximum concentration, the viscosity of a liquid is explained as mainly coming from the interparticle frictions of the solid particles.

Therefore, depending on the concentration of the particles in the suspension, the relative viscosity of plastic-modified bitumen can be calculated in three regimes: dilute regime, concentrated regime, and highly concentrated regime. In the first two regimes, the relative viscosity highly depends on the hydrodynamic behaviour of the suspension; however, at a high concentration, the frictional force between particles plays a more significant role.

**Figure 10:**
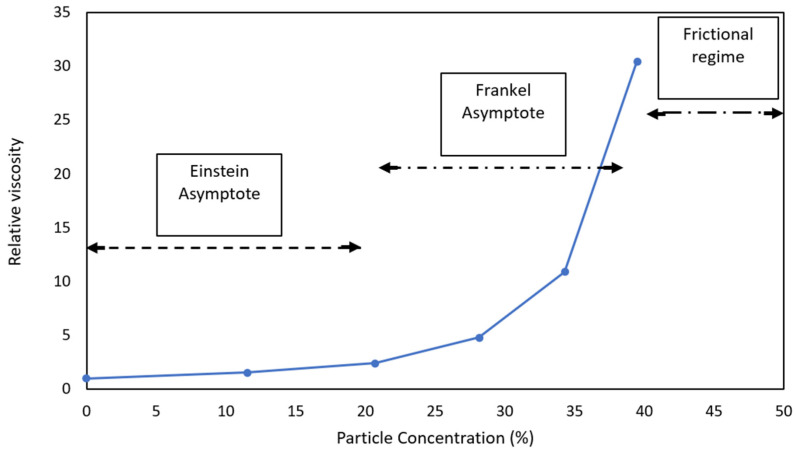
Relative viscosity at different concentration regimes.

To better illustrate this phenomenon, data from a study conducted by Zeng and Wu were extracted, as depicted in Figure 10 (the blue curve). They studied the effects of filler content on the viscosity of a binder [76]. Here, the authors marked the different regimes of the relative viscosity of a binder in a study conducted by Zeng and Wu. As shown, the Einstein Asymptote regime covers a 0% to 20% concentration, where viscosity is slightly increased by rising concentration. In the Frankel Asymptote regime, from 20% to around 40% concentration, viscosity sharply increases up to the point where viscosity mainly comes from interparticle friction.

In the case of dry-added plastics in the binder, the volume of the polymers after swelling is 1.15 to 3.54 times their virgin volume, as shown in Figure 6b. The enrichment of asphaltene in the matrix after the swelling of polymers can be explained using the following equation [43]:

(3) $$Asphaltene\;concentration\;enrichment\%=\left(\frac{\left(1-Polymer\;content\right)}{total\;volume\;of\;the\;binder-volume\;of\;PRP}-1\right)\times 100$$

**Figure 11:**
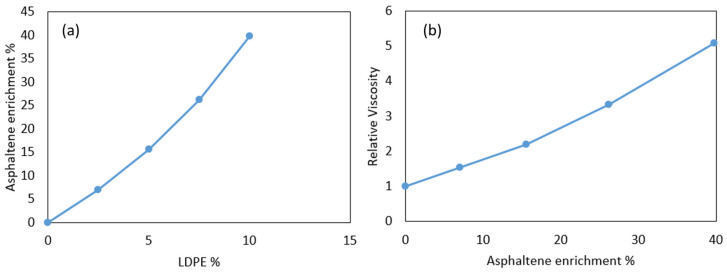
(**a**) The effects of LDPE inclusion in bitumen on asphaltene enrichment and (**b**) the effects of asphaltene enrichment of the matrix on binder viscosity. The swelling rate of LDPE is considered to be 3.54%, as calculated in Section 4.1. Dot points represent the test results, and the lines connect the test results to illustrate the trends.

To show the effect of asphaltene enrichment and its correlation with viscosity, a viscosity test was conducted on LDPE-modified binders with concentrations from 0 to 10% prepared via the wet process. The results are depicted in Figure 11.

The asphaltene enrichment is calculated using Equation (3), considering a swelling rate of 3.54% for LDPE. As shown in Figure 11a, 10% LDPE resulted in around 40% asphaltene enrichment, and given that asphaltenes typically make up 5–25% of the weight of bitumen [77], 10% LDPE can result in 7% to 35% asphaltene content. That increased asphaltene content contributes to raising the relative viscosity of the binder to more than 5, as shown in Figure 11b.

In order to maintain the viscosity of the matrix in a workable range, the total number of suspended particles in the suspension must remain constant. This can be accomplished by reducing the amount of filler added to the mixture. A higher asphaltene enrichment resulting from the incorporation of plastics necessitates a reduction in the amount of supplementary filler in the mixture. Therefore, when plastics are integrated into the asphalt mixture, they must be replacing a proportion of bitumen and filler. By reducing the filler content, the number of suspended particles in the plastic-modified binder reduces, potentially from the Frankle regime to the Einstein regime, and significantly decreases the overall viscosity of the binder, similar to that shown in Figure 10.

### 4.3. Marshall Tests and Air Voids

**Figure 12:**
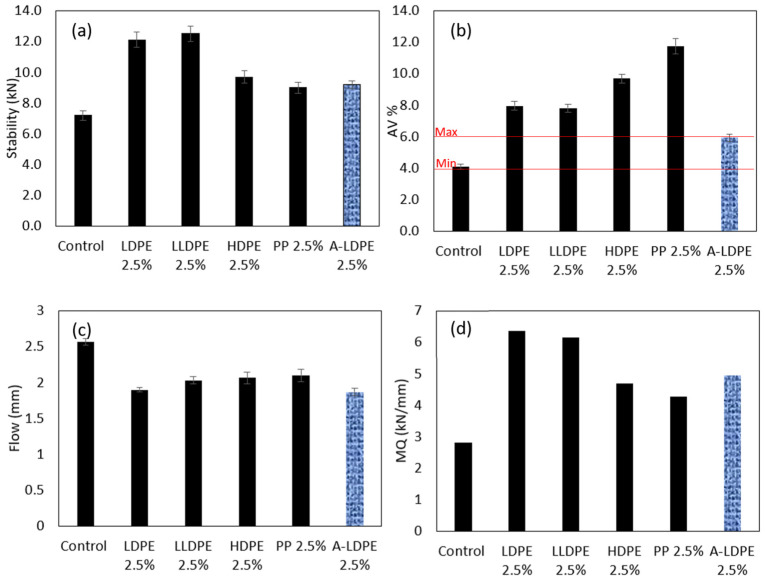
(**a**) Stability, (**b**) air voids, (**c**) flow, and (**d**) quotient of dry-added plastic-modified asphalt mixtures. Red lines represent specification limits.

To investigate the effects of modification on the mix design parameters of the asphalt mixture, Marshall stability and air void content were measured. The stability values and air void content of samples modified with various plastics are illustrated in Figure 12a,b, respectively. The stability of all modified samples increased, which aligns with expectations, as the binder’s viscosity was elevated, resulting in additional stiffness in the mixture. However, this increased stiffness reduced the workability of the binder, subsequently increasing the air void content, as demonstrated in Figure 12b. All types of plastics caused excessive AV in the asphalt mixture, higher than the maximum AV defined by Australian standards [54].

The sample modified with polypropylene (PP) exhibited the lowest workability, with an air void content of 11.8%. When compared to PP, the polyethylene samples displayed lower air void content and higher stability, indicating that asphalt modified with polyethylene offers superior workability and compactibility than asphalt modified with polypropylene. This is mainly due to the high melting point of PP compared with PEs.

Among the PE samples, both LDPE and LLDPE demonstrated higher stability and lower AV in comparison to HDPE. This is attributed to the higher swelling rate and homogeneity in LDPE and LLDPE due to their lower crystallinity when compared to HDPE.

The results of the stability and air void content tests revealed that the incorporation of 2.5% (by the total volume of the mixture) of plastics using the dry process significantly impacts the mix design of asphalt mixtures. This is due to the excessive viscosity of the binder matrix. To address this issue, the authors need to maintain the viscosity of the binder after polymer inclusion. To do so, filler content can be decreased to compensate for the asphaltene enrichment and excessive viscosity of the plastic-modified binder. To evaluate this hypothesis and its effects on asphalt parameters, an A-LDPE sample was made with reduced filler. In the A-LDPE sample, 50% (by volume) of the plastic is used as a substitute for the filler, and 50% (by volume) of it is used as a substitute for bitumen. LDPE was selected for this trial, and Marshall stability and AV were conducted on the samples. This amendment in the mix design led to a significant reduction in AV, which shows that the viscosity of the binder matrix decreased, and the workability of the asphalt mixture improved. It also led to lower stability, showing that the stiffness of the mixture reduced due to the amendment. The control sample and A-LDPE were the only mixes that met the range of AV (4% to 6%) [54] mentioned in the specifications.

The flow of the mixtures dropped due to the inclusion of plastics, as illustrated in Figure 12c. Generally, higher bitumen contents result in increased susceptibility to plastic flow, and as plastics replaced bitumen in the mixtures, lower flow was expected [78]. The reduction in flow can also be attributed to the higher viscosity of the binder matrix after the inclusion of plastics.

The Marshal Quotient of the mixtures, as an indicator of the bituminous mixture’s resistance to deformation, is depicted in Figure 12d. Both 2.5% LDPE and LLDPE significantly enhanced the resistance of the mixture against permanent deformation. All of the modified mixtures outperformed the control sample in this regard.

## 5. Conclusions

To understand the role of plastics in the mixture, it is necessary to know what occurs to the plastic particles from the moment they are added into the mixture to the time of compaction. It has been observed that the proper mixing of plastics with hot aggregate at a temperature of 180 °C, followed by subsequent mixing with hot bitumen, results in the complete integration of the plastics within the binder. Under these conditions, plastics do not remain as a proportion of the aggregates; instead, they become fully incorporated within the binder matrix.

The authors investigated asphaltene enrichment after plastic inclusion, the swelling rate of the polymers, and the variation of the dispersion of PRP in the matrix. This investigation drew the following conclusions based on the viscosity test, Marshall tests, AV, and morphological analyses of the samples:

→ The influence of dry-added plastics in an asphalt mixture varies depending on the method of introduction. However, the most effective approach is to initially mix plastics with hot aggregates at 180 °C (coat the aggregates) before introducing bitumen. This method ensures full integration of plastics into the binder matrix.
→ Higher plastic inclusion results in a lower swelling rate.
→ Improved dispersion of PRP throughout the binder was noted at a lower plastic inclusion level (1.25% by the total volume of the mixture).
→ The highest swelling rate, reaching 3.5, was observed in LDPE, attributed to its elevated amorphous content. Optimal dispersion of PRP within the matrix is likewise achieved with LDPE.
→ HDPE exhibits the lowest swelling rate.
→ The most heterogeneous matrix was obtained with the inclusion of dry-added polypropylene. This can be attributed to the elevated crystallinity of PP and its notably high melting point.
→ For dry-added modification, it is recommended to include plastic less than 2.5% by volume of the mixture.
→ Stability values and air void content vary among samples modified with different plastics, with polyethylene-modified samples exhibiting superior workability and compactibility compared to polypropylene-modified samples.
→ This study also suggests that using plastics to replace both bitumen and filler not only improves cost efficiency and reduces the carbon footprint but also enhances the overall performance of the asphalt mixture.
→ Incorporating 2.5% plastics impacts asphalt mixture design, resulting in excessive AV, which can be addressed by reducing the filler content of the mixture.
→ It is recommended to model the impact of plastic incorporation and subsequent filler substitution in future studies to guarantee the optimal mix design.
→ Further investigation is suggested to understand the effects of the modification on critical asphalt properties, especially air voids and rutting performance.

## Figures and Tables

**Table 1:** The role of waste plastics in the literature when added to the asphalt using the dry process.

Plastics	Plastic Shape	Aggregates Grading	Role in Mix Design	Reference
LDPE	Pellets	Constant	Aggregate	[15]
PET	Crushed	Variable	Aggregate	[16]
PE	flakes	Variable	Aggregate	[17]
Plastic	1–4 mm	Variable	Aggregate	[18]
LDPE, HDPE	Shredded	Constant	Bitumen modifier	[19]
PE, PP	1–6.3 mm	Constant	Filler	[20]
LDPE	Flakes	Constant	Not mentioned	[21]
LDPE, HDPE	Granules	Constant	Not mentioned	[22]
PET	Crushed	Constant	Not mentioned	[23]
PET	Crushed	Constant	Not mentioned	[24]
PET	Crushed	Constant	Not mentioned	[25]
PE	Shredded	Constant	Not mentioned	[26]
LDPE	Shredded	Constant	Coated aggregates	[27]
LDPE, HDPE	Shredded	Constant	Coated aggregates	[28]
PE	NM	Constant	Coated aggregates	[29]
Plastic	Shredded	Constant	Coated aggregates	[30]
Plastic	Shredded	Constant	Additive	[31]

**Table 2:** Characteristics of the plastics including melt flow rate (MFR), density, melting point, and crystallinity.

Plastics	MFR (g/10 min)	Density (g/cm^3^)	Melting Point (°C)	Crystallinity (%)
LDPE	21	0.917	106	20
LLDPE	1	0.921	121	50
PP	30	0.9	165	65
HDPE	20	0.956	127	70

**Table 3:** Asphalt mix designs for the evaluation of the role of the plastic in the dry process. Percentages are per volume of the total asphalt mixture. Samples prepared by Method 1 are labeled starting with ‘a’, while samples prepared by Method 2 are labeled starting with ‘b’.

Sample Labels	Mixing Method	Plastics (%)		Bitumen C170 (%)	Aggregates SMA10 (%)	Mixing Temperature (°C)
		PP	PE			
a-160	Method 1	1.25	1.25	12.65	84.85	160
a-180	Method 1	1.25	1.25	12.65	84.85	180
b-160	Method 2	1.25	1.25	12.65	84.85	160
b-180	Method 2	1.25	1.25	12.65	84.85	180

**Table 4:** Plastic contents of the mix designs by the total volume of the mixture used in the second step.

Run	Label	Plastic Type	Plastic Content (%)	Plastics/(Bitumen + Filler + Plastics) (%)
1	LDPE 1.25%	LDPE	1.25	4.74
2	LDPE 2.5%	LDPE	2.5	9.47
3	LLDPE 1.25%	LLDPE	1.25	4.74
4	LLDPE 2.5%	LLDPE	2.5	9.47
5	HDPE 1.25%	HDPE	1.25	4.74
6	HDPE 2.5%	HDPE	2.5	9.47
7	PP 1.25%	PP	1.25	4.74
8	PP 2.5%	PP	2.5	9.47
9	Control	-	0	0

**Table 5:** Plastic contents of the mix designs by the total volume of the mixture used in the third step.

Run	Label	Plastic Type	Plastic Content (%)	Amendment on Asphalt Mixture	Plastics/(Bitumen + Filler + Plastics) (%)
1	Control	-	0	Plastics replacing bitumen	0
2	LDPE 2.5%	LDPE	2.5	Plastics replacing bitumen	9.47
3	LLDPE 2.5%	LLDPE	2.5	Plastics replacing bitumen	9.47
4	HDPE 2.5%	HDPE	2.5	Plastics replacing bitumen	9.47
5	PP 2.5%	PP	2.5	Plastics replacing bitumen	9.47
6	A-LDPE 2.5%	LDPE	2.5	Plastics replacing filler and bitumen	9.47

**Table 6:** Marshall tests acceptable ranges based on Australian specifications.

Parameter	Units	Acceptable Range	Desirable Value	Reference
Stability	kN	>5.5	Peak point	[54]
Flow	mm	1.5–4	2–3.5	[54]
Air Voids (AV)	%	4–6	4–6	[54]

## Data Availability

Data are contained within the article.
